# Climatic suitability and future dynamics of test-weight-defined high-quality wheat in China under climate change

**DOI:** 10.3389/fpls.2026.1834560

**Published:** 2026-09-23

**Authors:** Yuncheng Zhao, Lei Zhang, Qing Li, Cong Zhang

**Affiliations:** 1 China Meteorological Administration (CMA) Henan Key Laboratory of Agrometeorological Support and Applied Technique, Henan, China; 2 National Meteorological Center, Beijing, China; 3 Haidian District Meteorological Bureau, Beijing, China; 4 Institute of Agricultural Economics and Technology, Hubei Academy of Agricultural Science, Wuhan, China

**Keywords:** China, climate change, climatic suitability, CMIP6, MAXENT model, test-weight-defined high-quality wheat

## Abstract

**Introduction:**

Climate change is expected to affect crop production and grain quality by altering the environmental conditions that define crop climatic niches. However, the spatial distribution and future dynamics of climatic suitability for test-weight-defined high-quality wheat in China remain insufficiently understood. This study aimed to characterize the climatic suitability of high-test-weight (High-TW) wheat and assess its potential changes under future climate scenarios.

**Methods:**

High-TW wheat was defined as wheat with a test weight (TW) ≥ 750 g L^-1^. Long-term wheat quality monitoring records, WorldClim bioclimatic variables, BCC-CSM2-MR-based future climate projections, and an optimized MaxEnt model were used to characterize the baseline suitability pattern, identify dominant climatic predictors, and project changes under four future climate scenarios.

**Results:**

The model showed strong discrimination under random holdout evaluation, with a mean test AUC of 0.936 and a continuous Boyce index of 0.9702. The minimum temperature of the coldest month (Bio6) was the dominant predictor, contributing 55.3% to the model, indicating the importance of winter thermal conditions in the climatic niche associated with High-TW wheat. Under the 1970–2000 baseline climate, high-suitability areas were mainly concentrated in the north-central Huang-Huai-Hai Plain, broadly corresponding to traditional production regions of strong- and medium-strong-gluten wheat. Under the BCC-CSM2-MR projections, the broad spatial pattern remained relatively stable, while changes occurred mainly in the composition and peripheral distribution of suitability classes. By 2081–2100, high-suitability area increased under all four scenarios, with the largest relative increase under SSP126 (+12.35%). Centroid shifts were modest and scenario-dependent rather than proportional to emission intensity, with the largest late-century displacement reaching approximately 48 km under SSP370.

**Discussion:**

Future climate change may primarily reorganize the internal structure of climatic suitability while maintaining the broad national-scale pattern of High-TW wheat suitability. These findings provide a national-scale climatic-suitability perspective for High-TW wheat zoning, regional adaptation, and future production-planning assessments in China.

## 1 Introduction

Wheat (*Triticum aestivum* L.) is one of the most widely cultivated cereal crops and an important staple food worldwide. In China, wheat contributes more than one-fifth of national grain production and plays a major role in food security (NBS, 2024). With rising living standards and changing dietary preferences, wheat production is increasingly expected to achieve both high yield and high quality. Wheat quality is often more sensitive than yield to climate variability and environmental conditions (Asseng et al., 2015). It is shaped by complex interactions between genotype and environment (G×E), which influence grain protein accumulation, starch synthesis, and processing characteristics (Williams et al., 2008). Climate change may increase wheat yield in some regions while reducing grain protein content or altering protein composition, indicating that wheat quality and yield do not necessarily respond to climatic change in the same way (Zahra et al., 2023; Xie and Yan, 2023). Studies in the North China Plain have also shown that wheat grain, flour, and dough traits contain clear climatic signals, highlighting the need to assess wheat quality from a climate-sensitive perspective (Zheng and Zhang, 2025).

The climatic effects on wheat quality are often nonlinear and depend on both the intensity and timing of climatic stress. Heat stress above 30°C during grain filling stage can restrict starch accumulation, accelerate crop development, and reduce grain test weight and processing quality (Gare et al., 2023). Water conditions also have complex effects. Moderate water deficit may promote grain protein accumulation, whereas severe drought can reduce photosynthesis, assimilate transport, and grain filling (Zhu et al., 2018). These event-scale processes motivate climate-sensitive assessment, but the present study characterizes long-term climatic suitability using bioclimatic summaries rather than directly modeling heat waves, frost events, short-term drought, or harvest-period rainfall. Changes in long-term temperature and precipitation regimes may alter the spatial distribution of areas climatically associated with High-TW wheat (Heikonen et al., 2025) (Porter and Semenov, 2005). Such information may contribute to broader production-zoning and adaptation assessments when considered together with non-climatic evidence. Previous studies have examined wheat-quality patterns using GIS, empirical thresholds, and statistical regression. Ma et al. (2021) identified regional differences in gain protein and wet gluten content (Ma et al., 2021). Strong- and medium-strong-gluten wheat production has traditionally been concentrated in the north-central North China Plain and the Hetao Irrigation Area (Zhao, 2010; Liu et al., 2024). Meteorological variables have also been increasingly integrated with wheat production or quality information to construct climatic suitability indices in China (Lv et al., 2023; Chen et al., 2024; Wang et al., 2024).

Ecological niche models provide an alternative approach for identifying nonlinear climatic constraints and projecting potential spatial changes under future climate conditions. MaxEnt is a presence-background model that hat estimates relative environmental suitability from occurrence records and predictors; background points represent available environments rather than true absences. Recent studies have used MaxEnt to assess wheat planting suitability and potential changes under climate warming (Ali et al., 2024; Guo et al., 2024). This approach is consistent with Hutchinson’s multidimensional niche theory, in which spatial distribution is constrained by the joint effects of multiple environmental variables (Colwell and Rangel, 2009). For cultivated crops, however, the predicted niche should be interpreted as a climatic-suitability envelope associated with observed production sites, not as a causal model or a probability of successful production.

Despite recent progress, the national-scale climatic suitability of test-weight-defined high-quality wheat remains insufficiently understood. Few studies have used long-term wheat-quality observations to identify climatic predictors associated with test-weight-defined quality and to examine the spatial configuration of suitable areas. Wheat quality is multidimensional, whereas spatially consistent records of protein, gluten, starch, and dough traits are not available for all regions and years. This study therefore focuses on test-weight-defined high-quality wheat (High-TW wheat), not comprehensive end-use quality. We hypothesized that its modeled climatic suitability would be associated with winter thermal conditions and growing-season hydrothermal factors, and that BCC-CSM2-MR-based future conditions would alter the composition and spatial configuration of relative suitability classes. The objectives were to (1) identify the national spatial pattern of climatic suitability for High-TW wheat under the 1970–2000 baseline, determine the dominant climatic drivers, and (2) project changes in relative suitability classes and centroid locations under four future climate scenarios. The results provide a climatic basis for high-quality wheat zoning, production planning, and adaptation to future climate change in China.

## 2 Materials and methods

### 2.1 Distribution data of test-weight-defined high-quality wheat

Wheat quality records were obtained from the annual *China Wheat Quality Report* published by the Ministry of Agriculture and Rural Affairs of China. The reports cover 2009-2022 (www.moa.gov.cn/, accessed on 15 April 2024). The dataset contained county-level records from major wheat-producing regions, including sampling locations, cultivars, and grain quality traits measured according to the national agricultural standard NY/T 3-1982. County level records were matched to the nearest national meteorological stations following the meteorological-station mapping method (Ma et al., 2021; Zhang et al., 2023). When multiple samples were available within the same county and year, their arithmetic mean was calculated to avoid overrepresentation repeatedly sampled location. Because records originated mainly from established wheat-production regions, their spatial pattern may reflect cultivar distribution, irrigation, management, soil conditions, regional production specialization, market orientation, and sampling intensity in addition to climate.

Occurrence points were defined as records with test weight (TW) ≥ 750 g L^−^¹ according to the Chinese national standard GB/T 17892-2024 (*High-quality Wheat*). This standard is used for first- and second-grade strong- and medium-gluten wheat. Records meeting this criterion were defined as test-weight-defined high-quality wheat, hereafter High-TW wheat, and were used as presence data. Test weight is a commercial indicator of grain density and filling; it does not encompass protein content, gluten strength, sedimentation value, starch characteristics, dough rheology, or end-use performance.

To reduce local spatial autocorrelation and repeated sampling from nearby environments, occurrence records were spatially thinned using a minimum distance of 10 km, with only one record retained when multiple records occurred within this distance (Aiello-Lammens et al., 2015). After quality screening, station matching, and spatial thinning, 439 occurrence points were retained for modeling (**Figure 1**). Spatial thinning reduces local clustering but does not eliminate residual spatial dependence or broad-scale sampling bias. A bias-corrected-background sensitivity analysis was therefore used to assess whether the principal modeled pattern was sensitive to the tested background-sampling scheme (Phillips et al., 2009).

**Figure 1:**
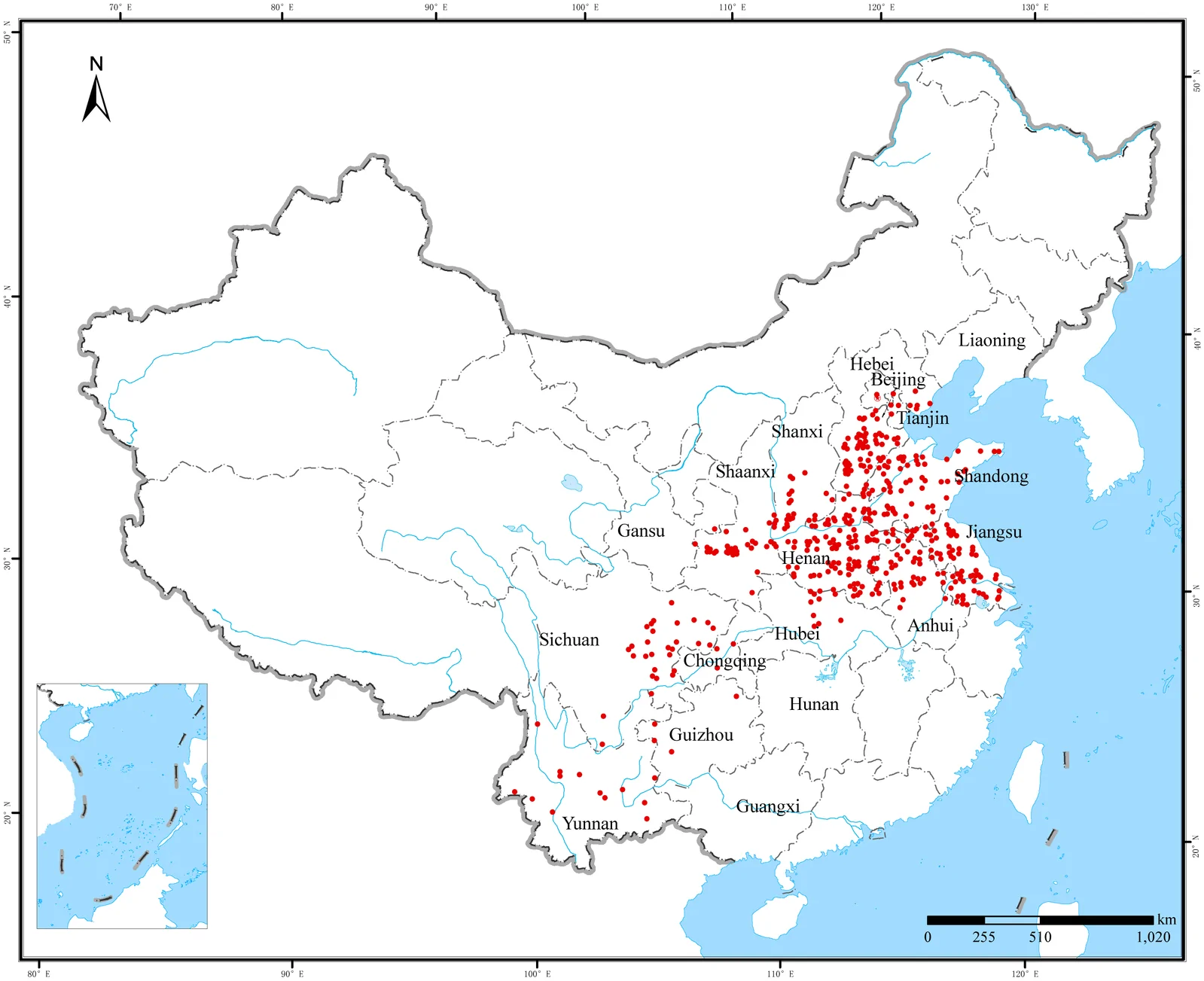
Geographic distribution of occurrence records for test-weight-defined high-quality wheat in China. The 439 occurrence records were defined using test weight ≥ 750 g L^−^¹ according to GB/T 17892–2024 and retained after spatial matching and 10 km spatial thinning.

### 2.2 Climate data acquisition and variable selection

A total of 19 bioclimatic variables (Bio1-Bio19) were used to characterize long-term temperature and precipitation regimes relevant to wheat growth and test-weight formation. These variables were obtained from the WorldClim database v2.1 (https://www.worldclim.org/data/cmip6/cmip6_clim2.5m.html, accessed on 26 October 2023) at a spatial resolution of 2.5 arc-minute resolution (approximately 5 km). The 1970–2000 climate normal was used as the reference baseline, with a 1991–2020 baseline-period sensitivity analysis described in Section 2.4. Future projections were obtained from the CMIP6 model BCC-CSM2-MR for SSP1-2.6, SSP2-4.5, SSP3-7.0, and SSP5-8.5 in 2041-2060 (mid-century) and 2081-2100 (the late century). BCC-CSM2-MR was developed by the Beijing Climate Center and has shown improved performance in simulating climate conditions over East Asia (Wu et al., 2019); it has also been used in habitat suitability studies in China (Fu et al., 2023). BCC-CSM2-MR was used as an internally consistent future-climate dataset and was not treated as representative of a full CMIP6 multi-model ensemble. Accordingly, all future results are conditional on BCC-CSM2-MR. All layers were aligned to a common 2.5 arc-minute WGS84 grid, clipped to the study area, and converted to ASCII format for MaxEnt (Zhao et al., 2024).

To reduce predictor redundancy, an initial MaxEnt model was fitted using all 19 bioclimatic variables provided preliminary screening information. Variable importance was evaluated using percent contribution and jackknife test, and variables contributing less than 1% were removed. Pearson correlation analysis was then calculated among the remaining variables. When two variables were strong correlated (|r| ≥ 0.8), the variable with the higher model contribution and clearer relevance to wheat growth and grain quality was retained (Carsten et al., 2013). This procedure resulted in four variables for the final model: Bio6, Bio8, Bio16, and Bio19 (**Table 1**). Final interpretation used complementary percent-contribution, permutation-importance, response-curve, and jackknife outputs, while recognizing their dependence on the fitted model and selected predictor set.

**Table 1:** Bioclimatic variables used for climatic niche modeling of high-quality wheat.

Code	Variable name	Unit
bio1	Annual Mean Temperature	°C
bio2	Mean Diurnal Range	°C
bio3	Isothermality (BIO2/BIO7) (×100)	–
bio4	Temperature Seasonality (standard deviation ×100)	–
bio5	Max Temperature of Warmest Month	°C
**bio6**	**Min Temperature of Coldest Month**	**°** **C**
bio7	Temperature Annual Range (BIO5-BIO6)	°C
**bio8**	**Mean Temperature of Wettest Quarter**	**°C**
bio9	Mean Temperature of Driest Quarter	°C
bio10	Mean Temperature of Warmest Quarter	°C
bio11	Mean Temperature of Coldest Quarter	°C
bio12	Annual Precipitation	mm
bio13	Precipitation of Wettest Month	mm
bio14	Precipitation of Driest Month	mm
bio15	Precipitation Seasonality (Coefficient of Variation)	–
**bio16**	**Precipitation of Wettest Quarter**	**mm**
bio17	Precipitation of Driest Quarter	mm
bio18	Precipitation of Warmest Quarter	mm
**bio19**	**Precipitation of Coldest Quarter**	**mm**

The bolded variables were used in modeling.

### 2.3 MaxEnt modeling and spatial analysis

The MaxEnt model (version 3.4.1; https://biodiversityinformatics.amnh.org/open_source/maxent/) was used to estimate relative climatic suitability for High-TW wheat under baseline and future conditions. The 439 occurrence records were used as presence data, while 10, 000 background points were randomly sampled from the study area to represent available environmental conditions rather than true absences. To reduce the risk of overfitting and to identify an appropriate level of model complexity, MaxEnt parameter tuning was conducted using the R package kuenm (Cobos et al., 2019). Candidate models comprised five feature-class combinations (L, LQ, H, LQH, and LQHP) and regularization multipliers of 0.5, 1.0, 1.5, 2.0, 3.0, and 4.0. All candidate models used the same occurrence records, background points, environmental variables, and evaluation settings. Model complexity was compared using the corrected Akaike information criterion (AICc) and delta AICc (Warren and Seifert, 2011). The combination with the lowest AICc was selected. RM = 1 with LQHP features was selected because it had the minimum AICc (9880.79; ΔAICc = 0; AICc weight = 0.9988) under the predefined minimum-AICc criterion (**Supplementary Figure 1**; **Supplementary Table 1**).

Ten replicate runs were conducted using the subsample method. In each run, 75% of occurrence records were randomly used for model training and the remaining 25% for testing. This random holdout evaluation was not a spatially independent or block cross-validation. The maximum number of iterations was set to 1, 000. Cloglog outputs provide continuous relative suitability values from 0 to 1. Response curves, percent contribution, permutation importance, and jackknife tests were used to describe model-derived associations and variable importance within the fitted model.

Suitability outputs were imported into ArcGIS 10.2 for spatial analysis. Continuous baseline suitability values were classified into marginal, low, moderate, and high suitability. Jenks natural breaks were applied once to the baseline suitability distribution to derive numerical class boundaries, and the same numerical thresholds were applied unchanged to every BCC-CSM2-MR future projection. Future scenarios were not independently reclassified using scenario-specific Jenks breaks. Classification was used only for visualization and area calculation and did not affect model calibration or continuous predictions. The classes are statistically defined relative-suitability categories, not physiologically or agronomically validated thresholds.

Future suitability changes were calculated by subtracting the baseline suitability map from future projections using the Raster Calculator tool. The SDMtoolbox was used to calculate the area of each suitability class and the geographic centroid of high-suitability areas (Jovanović et al., 2018). The centroid is a deterministic spatial summary of the mapped high-suitability class; displacement was interpreted as an approximate scenario-dependent indicator rather than a physiological migration process or a statistically estimated movement rate.

### 2.4 Model performance evaluation and sensitivity analyses

Model performance was evaluated across 10 replicate runs using mean training AUC, mean test AUC, AUC standard deviation, continuous Boyce index, 10th-percentile omission rate, and the mean training-test AUC difference (Hanley and McNeil, 1982); Hirzel et al., 2006. Test AUC was interpreted as discrimination between withheld occurrences and background environmental conditions under random holdout evaluation, not as general agronomic forecasting skill or spatially independent transferability. For the background-sampling sensitivity analysis, the randomBG reference was compared with a biasBG model. The bias surface was constructed on the common 2.5 arc-minute valid-cell grid from distance to the nearest occurrence using an exponential decay (σ=250 km) plus a strictly positive floor (1×10^−6^). Ten thousand background cells were sampled without replacement with probabilities proportional to those weights; this was a distance-decay occurrence-proximity surface, not a kernel-density surface. Continuous randomBG and biasBG maps were compared using raster correlation, upper-quartile overlap, and mean absolute difference.

To assess sensitivity to mismatch between the 2009–2022 occurrences and the 1970–2000 reference climate, an existing 1991–2020 comparison was incorporated. Monthly minimum temperature, maximum temperature, and precipitation for 1991–2020 were obtained from the WorldClim historical monthly weather dataset, based on CRU-TS 4.09 and downscaled and bias-corrected using WorldClim v2.1 (Fick and Hijmans, 2017; Harris et al., 2020). Thirty-year monthly climatologies were used to derive Bio6, Bio8, Bio16, and Bio19 following standard WorldClim definitions at 2.5 arc-minute resolution. The 1970–2000 and 1991–2020 randomBG models used the same 439 occurrences, 10, 000 background points, four predictors, RM = 1, LQHP features, cloglog output, and identical replicate and evaluation procedures. The comparison assessed sensitivity of performance and broad spatial patterns to climatological baseline selection; it was not interpreted as eliminating temporal mismatch (**Supplementary Table 3**).

## 3 Results

### 3.1 Model accuracy evaluation

Under random holdout evaluation, the optimized MaxEnt model showed good discrimination between withheld High-TW occurrence records and background environmental conditions under the 1970–2000 baseline, with a mean test AUC of 0.936 (**Figure 2**). The continuous Boyce index was 0.9702, the 10th-percentile omission rate was 0.1092, and the mean training-test AUC difference was 0.0014. Variation among replicate ROC curves was relatively small, indicting stable discrimination across replicate runs (Swets, 1988).

**Figure 2:**
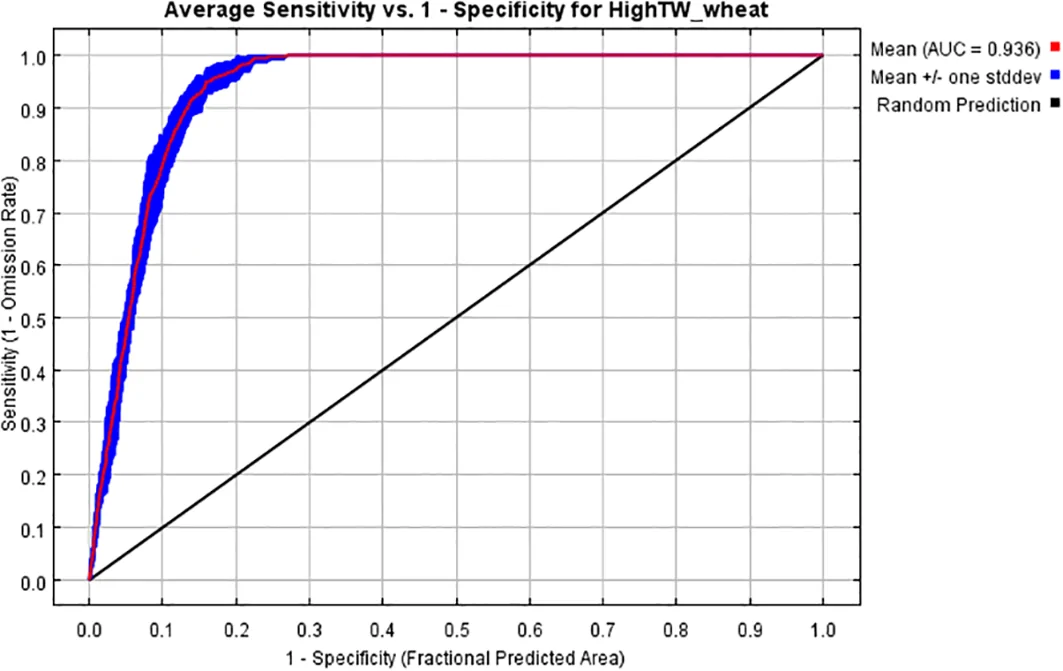
Mean receiver operating characteristic curve of the optimized MaxEnt model for High-TW wheat under the 1970–2000 baseline climate conditions. The red line represents the mean test ROC curve across replicate runs, the blue band represents one standard deviation, and the black diagonal line represents random prediction. The mean test AUC was 0.936.

Sensitivity analyses showed similar model performance and broad spatial patterns under alternative background sampling and climatological baselines. The 1970–2000 biasBG model had a test AUC of 0.9326, a continuous Boyce index of 0.9895, a 10th-percentile omission rate of 0.1141, and a training-test AUC difference of 0.0019. Its continuous suitability map showed high spatial consistency with the randomBG reference, with a raster correlation of 0.9791, an upper-quartile overlap of 0.7498, and a mean absolute difference of 0.0292 (**Supplementary Table 2**). For the baseline-period comparison, the 1970–2000 and 1991–2020 randomBG models had test AUC values of 0.9362 and 0.9352, continuous Boyce indices of 0.9702 and 0.9684, 10th-percentile omission rates of 0.1092 and 0.1095, and training-test AUC differences of 0.0014 and 0.0017, respectively. Their continuous suitability maps had a raster correlation of 0.9611, an upper-quartile overlap of 0.8361, and a mean absolute difference of 0.0219 (**Supplementary Table 3**). These results indicate that the principal national-scale suitability pattern was highly consistent between the two climatological baselines, supporting the use of the 1970–2000 baseline for the broad-scale analyses presented in this study.

### 3.2 Analysis of variable importance

Four bioclimatic variables played distinct roles in shaping the climatic niche distribution of High-TW wheat in China. Minimum temperature of coldest month (Bio6) was the most influential climatic predictor, with percent contribution rate=55.3% and permutation importance=58.9%) (**Figure 3**). These values describe predictive association within the selected MaxEnt model and do not demonstrate that Bio6 is a causal determinant.

**Figure 3:**
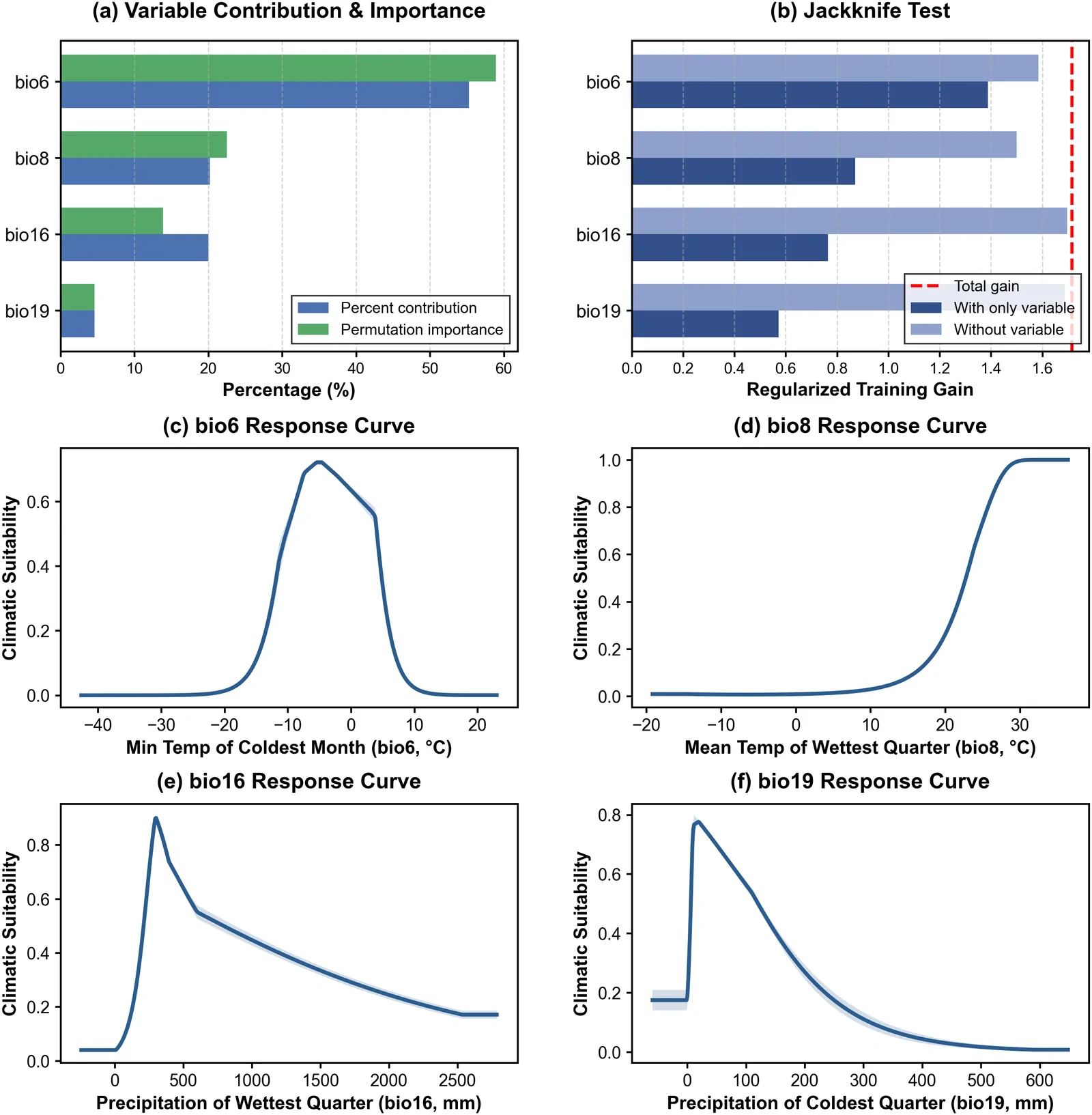
Relative importance and response curves of selected bioclimatic variables associated with the modeled climatic niche of High-TW wheat. **(a)** Percentage contribution and permutation importance of the four most influential bioclimatic variables. **(b)** Jackknife test of regularized training gain s. **(c–f)** Partial response curves for **(c)** Bio6, **(d)** Bio8, **(e)** Bio16, and **(f)** Bio19. The response ranges are model-derived associations, not validated physiological optima or biological thresholds.

The Jackknife test further indicated that Bio6 contained substantial model information: when used alone, it produced the highest training gain. Precipitation of the wettest quarter (Bio16) and mean temperature of the wettest quarter (Bio8) contributed 20.2% and 20%, respectively. Removing Bio8 caused a marked decline in model gain, indicating information not fully represented by the other selected predictors. These outputs remain conditional on the fitted predictor set.

Response curves revealed nonlinear, model-derived suitability associations. Modeled suitability was highest at approximately -5 to 5 °C for Bio6 and 200–500 mm for Bio16; it increased rapidly above approximately 20 °C for Bio8 and was highest at approximately 20–100 mm for Bio19. These ranges apply to partial responses within the fitted MaxEnt model and should not be interpreted as experimentally validated physiological optima, causal effects, or biological thresholds.

### 3.3 Spatiotemporal dynamics of suitable areas for high-quality wheat

During the 1970–2000 baseline, climatic suitability for High-TW wheat was centered on the Huang-Huai-Hai Plain (**Figure 4a**). Marginal, low, moderate, and high suitability areas accounted for 6.30%, 5.53%, 2.31%, and 3.01% of mainland China, respectively (**Figure 4b**). High- and moderate-suitability areas were mainly concentrated in north-central Henan, southern Hebei, western Shandong, northen Anhui, and parts of northern Jiangsu. Lower-suitability classed extended along western, southern, and southwestern transition zones.

**Figure 4:**
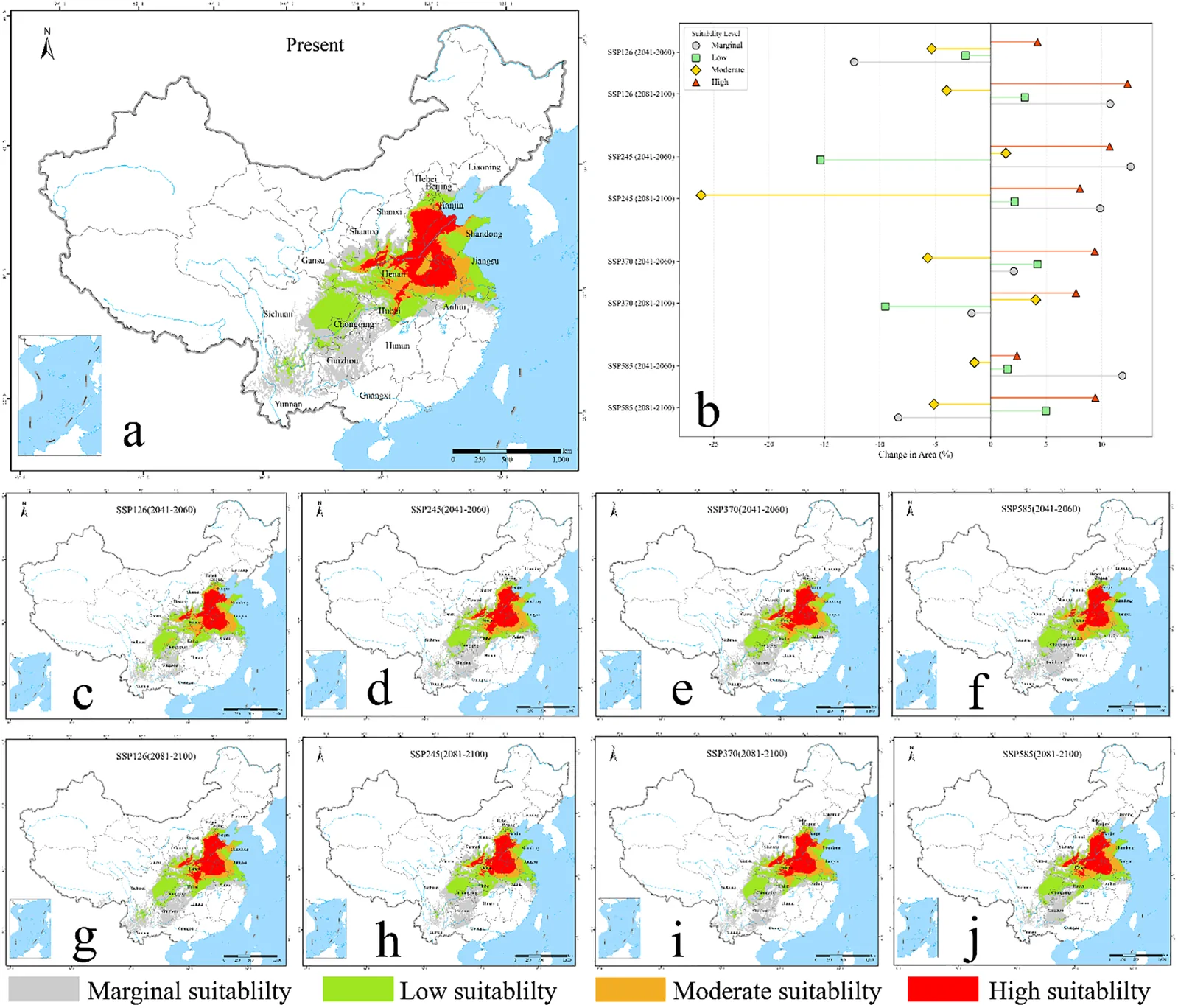
BCC-CSM2-MR-based changes in climatic suitability of High-TW wheat. **(a)** Baseline distribution (1970-2000). **(b)** Relative area changes for marginal, low, moderate, and high suitability under SSP126, SSP245, SSP370, and SSP585 in 2041–2060 and 2081-2100; positive values indicate increases relative to baseline. **(c–f)** Mid-century maps and **(g–j)** late-century maps, ordered by SSP126, SSP245, SSP370, and SSP585.

Under the BCC-CSM2-MR projections, the broad spatial pattern remained similar to the baseline, with the main high-suitability region continuing to be centered on the Huang-Huai-Hai Plain (**Figure 4**). In absolute terms, high-suitability area increased from 28.47×10^4^ km² at baseline to 31.98, 30.76, 30.66, and 31.16×10^4^ km² under late-century SSP126, SSP245, SSP370, and SSP585, respectively. Under SSP126, high suitability increased by 4.23% during 2041–2060 and 12.35% during 2081–2100 relative to baseline. Under SSP245, high suitability increased by 10.73% and 8.06%, respectively, while late-century moderate suitability declined by 26.16%. Under SSP370, high suitability increased by 9.40% and 7.70%; under SSP585, it increased by 2.38% and 9.45%. SSP126 therefore showed the largest modeled late-century relative increase among the BCC-CSM2-MR scenarios examined here (**Figures 4c–j**). A tentative model-based interpretation is that moderate warming may move some cold-limited locations toward the composite conditions associated with current High-TW occurrences, but this ranking is scenario- and GCM-dependent and should not be generalized as a benefit of low emissions for wheat quality.

Overall, the combined proportion of the four suitability classes ranged from approximately 16.23% to 18.28% across future scenarios, compared with 17.15% during the baseline period. The projections mainly showed internal and peripheral redistribution rather than a fundamental national-scale shift.

### 3.4 Spatial shift of the centroid of high suitability areas

Under baseline climate conditions, the centroid of the high-suitability class was located in the central Huang-Huai-Hai Plain (115.32°E, 35.37°N). The projected centroid trajectories differed among climate scenarios, although the late-century centroids were generally located west or southwest of the baseline centroid (**Figure 5**). During 2041-2060, the centroid shifted approximately 19 km eastward under SSP1-2.6, 29 km southwestward under SSP2-4.5, 38 km westward under SSP3-7.0, and 17 km east-northeastward under SSP5-8.5. During 2081-2100, the corresponding displacem ents from the baseline centroid were approximately 26, 37, 48, and 26 km, respectively. The largest modeled late-century displacement occurred under SSP3-7.0. The SSP1-2.6 and SSP5-8.5 trajectories showed directional reversals between the mid- and late-century periods, whereas SSP2-4.5 and SSP3-7.0 showed predominantly westward or southwestward adjustments. Overall, the projected centroid shifts were modest and scenario-dependent rather than increasing consistently with emission intensity.

**Figure 5:**
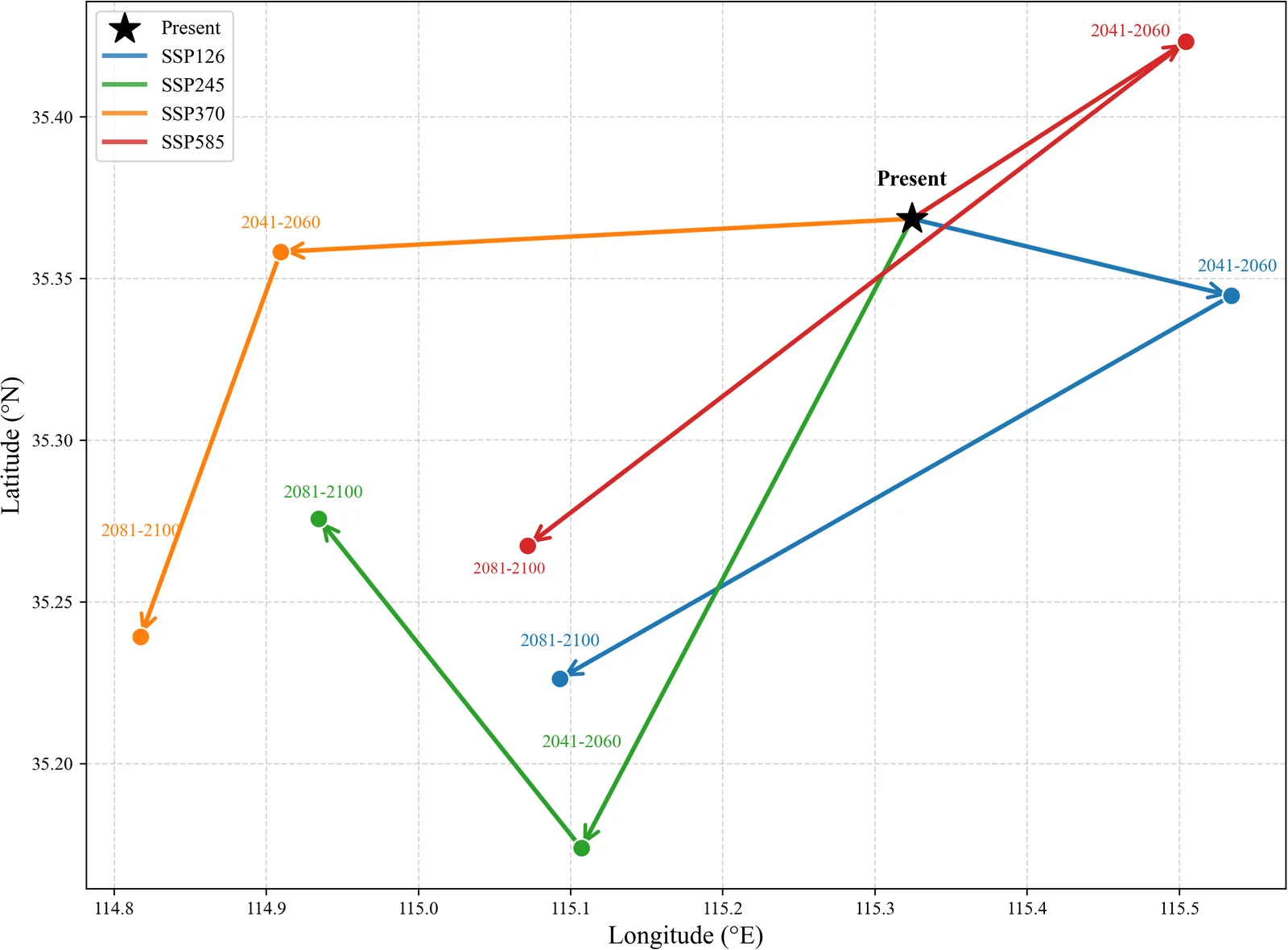
Projected centroid shifts of the high-suitability area for high-TW wheat under the BCC-CSM2-MR scenarios.

## 4 Discussion

### 4.1 Applicability of climatic niche modeling for wheat quality

The optimized MaxEnt model showed consistent discrimination under random holdout evaluation, with a mean test AUC of 0.936, a continuous Boyce index of 0.9702, and a small training-test AUC difference of 0.0014. These results indicate that the selected bioclimatic variables captured broad climatic characteristics associated with the observed distribution of High-TW wheat distributon. The high spatial correlation between the random-background and bias-corrected-background models (r = 0.9791) further indicates that that the broad suitability pattern was relatively stable under the two background-sampling schemes tested. However, AUC in presence-background models is influenced by the extent and environmental characteristics of the background and should not be interpreted as a direct measure of agronomic predictive accuracy (Lobo, 2008). Although 10 km spatial thinning reduced local clustering, the random training-testing partition did not provide fully spatially independent validation, and the reported evaluation metrics may therefore remain somewhat optimistic with respect to spatial transferability. MaxEnt estimates relative environmental suitability from occurrence and background data rather than true absences, which is appropriate where High-TW production sites are known but the status of unsampled environments is uncertain (Phillips et al., 2006).

It should also be emphasized that the suitability values generated by MaxEnt do not represent the probability of successful wheat production in a strict agronomic sense. Rather, they indicate the relative degree to which local climatic conditions resemble those associated with known High-TW wheat sites. In this study, the occurrence records were defined using test weight according to GB/T 17892-2024. Therefore, the modeled climatic niche should be interpreted as the climatic suitability of test-weight-defined high-quality wheat rather than as a comprehensive representation of all dimensions of wheat quality. This distinction is important because wheat quality also involves protein content, gluten strength, starch properties, dough rheology, and end-use performance. In addition, the occurrence records were mainly derived from established wheat-producing regions, and their spatial distribution may also reflect cultivar choice, irrigation, field management, soil conditions, regional production specialization, and sampling intensity. A presence-background model cannot fully separate these influences from climatic associations. Accordingly, the present model does not describe realized production outcomes directly but identifies a broad climatic envelope associated with the observed distribution of High-TW wheat. In this regard, climatic niche modeling offers a useful framework for examining the climatic dimension of crop-quality distribution and national scales (Bradie and Leung, 2017).

### 4.2 Key climatic variables shaping the climatic niche of high-quality wheat

Among the selected predictors, the minimum temperature of the coldest month (Bio6) showed the strongest association with the model climatic niche of High-TW wheat, suggesting that cold-season thermal conditions are an important component of the climatic environment associated with its observed distribution. Although Bio6 represents winter thermal conditions rather than a direct grain-filling variable, winter temperature can influence overwintering, vernalization progression, and subsequent spring phenological development, which may further affect developmental timing, grain-filling duration, source-sink relationships, assimilate transport, starch accumulation, and ultimately grain density and test weight (Asseng et al., 2015; (Chen et al., 2024; Dupont and Altenbach, 2003). Growing-season and seasonal hydrothermal conditions represented by Bio8, Bio16, and Bio19 also contributed substantially to the modeled climatic niche. Suitable combinations of temperature and moisture are favorable for grain filling and assimilate accumulation, whereas excessive heat or unfavorable water conditions can shorten grain-filling duration, inhibit starch synthesis, alter assimilate partitioning, and reduce grain density and other quality characteristics (Cao et al., 2024) (Hurkman et al., 2003; Ben Mariem et al., 2021). The response curves futher showed nonlinear associations between climatic conditions and modeled suitability, with relatively high predicted suitability occurring at approximately -5 to 5 °C for Bio6 and 200–500 mm for Bio16, together with corresponding high-suitability ranges for Bio8 and Bio19. These ranges represent model-derived suitability associations rather than experimentally established physiological optima or biological thresholds (Zhou et al., 2025). Overall, the results indicate that the climatic niche of High-TW wheat is associated with a multidimensional climatic environment involving both cold-season thermal conditions and growing-season hydrothermal regimes, which is broadly consistent with the study hypothesis. However, the relative importance of these variables represents predictive association rather than direct physiological causation, and the modeled niche should be interpreted as a climate-related suitability envelope associated with the observed distribution of High-TW wheat rather than a fully realized production niche, because local test-weight outcomes may also be modified by cultivar characteristics, irrigation, nutrient and field management, soil conditions, and other production factors.

### 4.3 Changes in suitable areas under different climate scenarios

Under baseline climate, climatically suitable areas for high-quality wheat were mainly concentrated in the north-central Huang-Huai-Hai Plain, broadly corresponding to traditional production regions of strong- and medium-strong-gluten wheat in China. This spatial pattern may be related to the combination of relatively favorable winter thermal conditions and suitable hydrothermal conditions during subsequent wheat development grain filling (Xu et al., 2024). Under the BCC-CSM2-MR projections, the core high-suitability region remained centered on the Huang-Huai-Hai Plain, while the proportions and peripheral distributions of different suitability classes varied among scenarios and periods. The combined proportion of the four suitability classes ranged from approximately 16.23% to 18.28% across the future scenarios, compared with 17.15% during the baseline climate. These results suggest that, within the BCC-CSM2-MR projections examined here, future climatic change is more likely to modify transitions among suitability classes and their peripheral distribution than to cause a fundamental national-scale redistribution of the climatic suitability pattern.

Changes in individual suitability classes were scenario-depended and did not increase monotonically with emission intensity. By 2081-2100, the high-suitability areas increased under all four scenarios, with the largest relative increase under SSP126 (+12.35%), followed by SSP585 (+9.45%), SSP245 (+8.06%), and SSP370 (+7.70%). Under SSP245, the moderate-suitability area declined by 26.16% during 2081-2100, indicating marked redistribution among adjacent suitability classes (Porter and Semenov, 2005). Under SSP370, the high-suitability areas increased by 9.40% during 2041–2060 and by 7.70% during 2081-2100. One possible explanation for the non-monotonic response is that moderate warming may shift some currently cold-limited locations toward climatic conditions more similar to those associated with observed High-TW wheat, whereas stronger warming together with changes in seasonal precipitation may shift other areas away from these conditions (Zhang et al., 2023). Therefore, warming should not be interpreted as uniformly beneficial for test-weight-based climatic suitability (Zhao et al., 2017). Excessive warming can shorten grain-filling duration, intensify heat stress, alter assimilate accumulation, and adversely affect grain density and other quality traits (Kong et al., 2023). However, the different responses among SSPs identified here are conditional on BCC-CSM2-MR and should not be interpreted as a general physiological response to emission intensity. In addition, because suitability classes were defined using fixed baseline-derived statistical thresholds, changes in class area should be interpreted as relative redistribution within the modeled suitability surface rather than as shifts across biologically validated quality thresholds.

Centroid trajectories also differed among scenarios rather than showing a consistent increase in displacement with emission intensity. By 2081-2100, the centroids were generally located west or southwest of the baseline position, and the largest modeled displacement was approximately 48 km under SSP370. Directional reversals under SSP126 and SSP585 further indicate that the projected changes represent scenario-dependent spatial adjustment rather than uniform migration of the entire suitable region. Together with the relatively stable core distribution, these results suggest that climate change may primarily reorganize the internal spatial configuration of climatically suitable areas while maintaining the Huang-Huai-Hai Plain as the principal high-suitability region under the BCC-CSM2-MR projections (Ali et al., 2024). From an adaptation perspective, the modeled changes may help identify areas where adjustments in cultivar choice, sowing-date optimization, and water and nutrient management warrant further agronomic evaluation (Zhao et al., 2025). Such projections should nevertheless be regarded as one climatic component of adaptation planning rather than a direct prescription for future production layout, because realized wheat quality and production potential also depend on cultivar, soil, irrigation, management, and socioeconomic conditions.

### 4.4 Limitations and future perspectives

This study provides a national-scale assessment of climatic suitability for test-weight-defined high-quality wheat, but several limitations remain. First, the 2009–2022 occurrence records and the 1970–2000 climatological baseline are not fully contemporaneous. Nevertheless, the additional 1991–2020 sensitivity analysis produced very similar model performance and broad spatial patterns, with a raster correlation of 0.9611, an upper-quartile overlap of 0.8361, and a mean absolute difference of 0.0219, indicating that the principal national-scale suitability pattern was relatively robust to baseline-period selection. Second, future projections were based on BCC-CSM2-MR and therefore represent scenario responses conditional on this GCM rather than the full range of CMIP6 uncertainty. Third, the model focuses specifically on test weight and long-term climatic conditions. Test weight represents an important commercial grain-quality trait but does not encompass all dimensions of wheat quality, while long-term bioclimatic variables cannot fully represent short-duration weather stresses or the effects of cultivar, soil, irrigation, and field management.

Despite these limitations, the combination of long-term quality observations, optimized MaxEnt parameterization, complementary model-evaluation metrics, spatial thinning, background-sampling sensitivity analysis, and baseline-period sensitivity analysis provides a consistent framework for identifying broad climatic associations and spatial patterns of High-TW wheat at the national scale. The results are therefore most appropriately interpreted as a climatic-suitability assessment rather than a direct prediction of realized production or comprehensive wheat quality. Future studies could further extend this framework by integrating multiple quality traits, temporally matched climate observations, phenology-specific and extreme-weather indicators, spatially structured validation, multi-GCM ensembles, and field or agronomic information, thereby improving both mechanistic understanding and practical application.

## 5 Conclusion

This study identified the national-scale climatic suitability pattern and future spatial dynamics of test-weight-defined high-quality wheat in China using an optimized MaxEnt model. The minimum temperature of the coldest month (Bio6) was the dominant climatic predictor, with a contribution of 55.3%, indicating the importance of winter thermal conditions in the climatic niche associated with High-TW wheat. Under the 1970–2000 baseline, high-suitability areas were mainly concentrated in the north-central Huang-Huai-Hai Plain. Under the BCC-CSM2-MR projections, future climate change is expected to modify the composition and peripheral distribution of suitability classes rather than fundamentally alter this broad spatial pattern. High-suitability areas increased under all four scenarios by the late century, with the largest relative increase occurring under SSP126 (+12.35%). Centroid shifts were scenario-dependent rather than proportional to emission intensity, and the largest late-century displacement occurred under SSP370, reaching approximately 48 km. Overall, the results suggest that future climate change may primarily reorganize climatic suitability within and around existing wheat-producing regions while maintaining Huang-Huai-Hai Plain as the principal high-suitability region. These findings provide a national-scale climatic-suitability perspective for High-TW wheat zoning, targeted regional adaptation, and future production-planning assessments in China, while also providing a basis for integrating climatic information with cultivar, management, soil, and water-resource conditions in subsequent applications.

## Data Availability

The raw data supporting the conclusions of this article will be made available by the authors, without undue reservation.
