# Supplementary material for: Climatic suitability and future dynamics of test-weight-defined high-quality wheat in China under climate change
Source: Front Plant Sci. 2026 Sep 23;17:1834560. doi: 10.3389/fpls.2026.1834560 (PMC13642596; doi:10.3389/fpls.2026.1834560)
Supplement: Supplementary file 1 [file DataSheet1.docx]

Supplementary Material

# 1 Supplementary Figures and Tables

## 1.1 Supplementary Figures

**Supplementary Figure 1. MaxEnt parameter tuning based on ΔAICc across regularization multipliers and feature-class combinations.** Lower ΔAICc values indicate stronger model support. Candidate models included five feature-class combinations (L, LQ, H, LQH, and LQHP) and regularization multipliers ranging from 0.5 to 4.0. The model with RM = 1 and FC = LQHP had the lowest AICc value (ΔAICc = 0) and was selected for the final suitability projections.

## 1.2 Supplementary Tables

**Supplementary Table 1** **AICc-based comparison of the best-supported regularization multiplier within each feature-class combination evaluated during MaxEnt parameter tuning.**

| **Feature** | **Best RM** | **AICc** | **ΔAICc** | **AICc weight** | **Mean test AUC** | **Omission rate** |
| --- | --- | --- | --- | --- | --- | --- |
| L | 1.0 | 10748.52 | 867.73 | 3.75×10⁻¹⁸⁹ | 0.8652 | 0.1049 |
| LQ | 0.5 | 9908.57 | 27.78 | 9.27×10⁻⁷ | 0.9320 | 0.1003 |
| H | 1.5 | 9986.08 | 105.29 | 1.37×10⁻²³ | 0.9372 | 0.1071 |
| LQH | 1.0 | 9895.24 | 14.45 | 7.26×10⁻⁴ | 0.9377 | 0.1048 |
| **LQHP** | **1.0** | **9880.79** | **0.00** | **0.9988** | **0.9359** | **0.1049** |

**Note:** Candidate models included five feature-class combinations (L, LQ, H, LQH, and LQHP) and regularization multipliers from 0.5 to 4.0. For each feature-class combination, the model with the lowest AICc is shown. The overall best-supported model was RM = 1 with LQHP features, which had the minimum AICc (9880.79), ΔAICc = 0, and an AICc weight of 0.9988. Model selection was based on AICc rather than AUC alone to balance goodness of fit and model complexity.

**Supplementary Table 2** **Sensitivity analysis of model performance and spatial consistency under different background sampling schemes for the 1970–2000 baseline climate.**

| **Model** | **Baseline period** | **Background scheme** | **Mean test AUC** | **Boyce index** | **10% omission rate** | **Train–test ΔAUC** | **Raster r** | **Upper-Q overlap** | **MAD** |
| --- | --- | --- | --- | --- | --- | --- | --- | --- | --- |
| randomBG | 1970–2000 | Random background | 0.9362 | 0.9702 | 0.1092 | 0.0014 | Reference | Reference | 0.0000 |
| biasBG | 1970–2000 | Bias-corrected background | 0.9326 | 0.9895 | 0.1141 | 0.0019 | 0.9791 | 0.7498 | 0.0292 |

**Note:** The 1970–2000 randomBG model was used as the reference model for both the background-sampling sensitivity analysis and the baseline-period sensitivity analysis reported in Supplementary Table 3. The randomBG and biasBG models used the same occurrence records, environmental predictors, replication procedure, and optimized MaxEnt settings (RM = 1 and FC = LQHP). Raster correlation and mean absolute difference were calculated from the continuous suitability outputs. Upper-quartile overlap was calculated as the intersection over union of the areas within the highest 25% of suitability values. Background points represent the environmental conditions available within the study area and should not be interpreted as true absence records.

**Supplementary Table 3. Sensitivity analysis comparing model performance and spatial consistency under the 1970–2000 and 1991–2020 climatological baselines.**

| **Model** | **Background scheme** | **Mean test AUC** | **Boyce index** | **10% omission rate** | **Train–test ΔAUC** | **Raster r** | **Upper-Q overlap** | **MAD** |
| --- | --- | --- | --- | --- | --- | --- | --- | --- |
| BIO(1970-2000)-randomBG | Random background | 0.9362 | 0.9702 | 0.1092 | 0.0014 | Reference | Reference | 0.0000 |
| BIO(1991-2020)-randomBG | Random background | 0.9352 | 0.9684 | 0.1095 | 0.0017 | 0.9611 | 0.8361 | 0.0219 |

**Note:** T The 1970–2000 randomBG model was the same reference model used in the background-sampling sensitivity analysis reported in Supplementary Table 2. The 1970–2000 and 1991–2020 models used the same 439 occurrence records, 10,000 random background points, Bio6, Bio8, Bio16, and Bio19, RM = 1, LQHP features, cloglog output, and identical replicate and evaluation procedures. Raster correlation and mean absolute difference were calculated from the continuous suitability outputs, using the 1970–2000 randomBG model as the reference. Upper-quartile overlap was calculated as the intersection over union of the areas within the highest 25% of suitability values. This comparison assesses sensitivity to climatological baseline selection and does not eliminate the temporal mismatch between occurrence observations and the reference climate period..
